# Molecular Species Delimitation in the *Racomitrium canescens* Complex (Grimmiaceae) and Implications for DNA Barcoding of Species Complexes in Mosses

**DOI:** 10.1371/journal.pone.0053134

**Published:** 2013-01-14

**Authors:** Michael Stech, Sarina Veldman, Juan Larraín, Jesús Muñoz, Dietmar Quandt, Kristian Hassel, Hans Kruijer

**Affiliations:** 1 Naturalis Biodiversity Center, Section National Herbarium of the Netherlands, Leiden University, Leiden, The Netherlands; 2 Departamento de Botánica, Universidad de Concepción, Concepción, Chile; 3 Real Jardín Botánico, Consejo Superior de Investigaciones Científicas (RJB-CSIC), Madrid, Spain; 4 Centro de Investigación de la Biodiversidad y Cambio Climático, Universidad Tecnológica Indoamérica, Quito, Ecuador; 5 Nees-Institute for Plant Biodiversity, Rheinische Friedrich-Wilhelms-Universität, Bonn, Germany; 6 Museum of Natural History and Archaeology, Norwegian University of Science and Technology, Trondheim, Norway; University of Oxford, United Kingdom

## Abstract

In bryophytes a morphological species concept is still most commonly employed, but delimitation of closely related species based on morphological characters is often difficult. Here we test morphological species circumscriptions in a species complex of the moss genus *Racomitrium*, the *R. canescens* complex, based on variable DNA sequence markers from the plastid (*rps4-trnT-trnL* region) and nuclear (nrITS) genomes. The extensive morphological variability within the complex has led to different opinions about the number of species and intraspecific taxa to be distinguished. Molecular phylogenetic reconstructions allowed to clearly distinguish all eight currently recognised species of the complex plus a ninth species that was inferred to belong to the complex in earlier molecular analyses. The taxonomic significance of intraspecific sequence variation is discussed. The present molecular data do not support the division of the *R. canescens* complex into two groups of species (subsections or sections). Most morphological characters, albeit being in part difficult to apply, are reliable for species identification in the *R. canescens* complex. However, misidentification of collections that were morphologically intermediate between species questioned the suitability of leaf shape as diagnostic character. Four partitions of the molecular markers (*rps4-trnT*, *trnT-trnL*, ITS1, ITS2) that could potentially be used for molecular species identification (DNA barcoding) performed almost equally well concerning amplification and sequencing success. Of these, ITS1 provided the highest species discrimination capacity and should be considered as a DNA barcoding marker for mosses, especially in complexes of closely related species. Molecular species identification should be complemented by redefining morphological characters, to develop a set of easy-to-use molecular and non-molecular identification tools for improving biodiversity assessments and ecological research including mosses.

## Introduction

Mosses (Bryophyta) represent the most species rich of the three lineages of bryophytes, and the second most species rich lineage of land plants after angiosperms [1]. Mosses contribute significantly to the biodiversity of terrestrial ecosystems. For example, species of the moss genus *Racomitrium* s.l. (Grimmiaceae, Bryophyta) are important components of many terrestrial and saxicolous habitats, from coastal sand dunes to mountain ecosystems to the arctic tundra. However, ecological and biodiversity research aiming at including bryophytes is often hampered by unclear species circumscriptions and identification difficulties of bryophyte taxa based on morphological characters. This is especially true for complexes of closely related, morphologically similar species.

In bryophytes, a morphological species concept is still most commonly employed, i.e., species are groups of individuals or populations that are morphologically distinguishable from other groups [1]. DNA sequence analyses allow testing the morphological species circumscriptions and providing new insights into species relationships. An increasing number of studies of bryophyte species, especially moss species, has already revealed incongruence between morphological species circumscriptions and molecular data (see [1], [2] for review). In particular, heterogeneity between rates of molecular versus morphological evolution seems to be evident in bryophytes, partly leading to a hidden molecular diversity and cryptic speciation (e.g. [3], [4]). DNA sequence analyses can provide new tools for species identification as well by comparing sequences from unidentified specimens with a reference database of sequences of identified specimens (DNA barcoding), which can also be used by researchers not specialized in bryology. Although *rbcL* and *matK* were advocated as core DNA barcoding markers for land plants [5], the identification success using these two markers has been demonstrated to be below 70% in angiosperms (e.g. [6]). Therefore, other potential barcoding markers need to be tested and discussed, especially for bryophytes (e.g. [7]–[11]), which have lower molecular rates compared to angiosperms [12].

The taxonomy of the genus *Racomitrium* exemplifies the need of testing morphological taxon circumscriptions by molecular data. Morphologically distinguishable groups have been treated either at different taxonomic levels within a broadly defined genus *Racomitrium*, or as four separate genera [13], which led to considerable changes in species names. For example, the *R. canescens* species complex was treated as genus *Niphotrichum* [13]. First molecular phylogenetic reconstructions, however, supported a monophyletic *Racomitrium* clade [14], [15].

The *Racomitrium canescens* complex is of Holarctic distribution and widespread in most parts of the northern temperate to arctic zones. It is easily distinguished from the other *Racomitrium* species by a combination of morphological characters, which include strongly papillose laminal cells with the tall papillae situated over the cell lumina, very long peristome teeth that are regularly cleft to base in 2–3 filiform prongs, and hyaline alar cells forming often decurrent auricles. The extensive morphological variability within the complex has led to different opinions in the older literature about the number of species and intraspecific taxa that should be distinguished (cf. [16], [17]). Consequently, Frisvoll [17] aimed at finding stable morphological characters that should represent different species, and distinguishing these characters from environmental modifications. Based mainly on leaf characters (leaf shape, hairpoint morphology, nerve length, alar cells and basal marginal leaf cells), Frisvoll [17] carried out a comprehensive taxonomic revision, in which eight species in two subsections were recognised, a classification still accepted to date (e.g. [18]). Frisvoll [17] argued that his morphologically defined species represent different genotypes, due to the frequent occurrence of mixed populations of two or more types of morphologically distinguishable plants (for diagnostic morphological characters of the species see [17], [18]).

Four species are widespread across the Holarctic, namely *R. canescens* s.str., *R. elongatum*, *R. ericoides*, and *R. panschii*, the latter being mainly confined to the Arctic, whereas the other four species occur mainly in western North America (*R. pygmaeum*, *R. muticum*) or East Asia (*R. barbuloides*, *R. japonicum*), respectively. In addition, Frisvoll [17] distinguished two subspecies within *R. canescens* s.str., subsp. *canescens* and subsp. *latifolium*. They are found in mixed populations in an overlapping part of their otherwise separated distribution areas, but are morphologically intergrading and were therefore not considered separate species by Frisvoll [17].

The available taxonomic revisions and floristic treatments (e.g. [16]–[18]) provided a sound basis for testing morphological species circumscriptions in the *R. canescens* complex. Molecular phylogenetic reconstructions supported the monophyly of the complex [15], [19] and indicated that another species, the western North American *R. varium*, belongs to the complex as well [15]. The latter was considered to belong to the *Racomitrium* segregate *Codriophorus* [13], which differs from *Niphotrichum* by having leaf papillae situated over the cell walls, undifferentiated or coloured alar cells, and shorter peristome teeth. In *R. varium*, however, the peristome teeth are long, which supports its position in the *R. canescens* complex. A clade composed of two species, *R. fasciculare* and *R. laevigatum*, was resolved as sister group of the *R. canescens* complex [15], [19]. A first case study to evaluate molecular markers for species identification in Grimmiaceae indicated that DNA barcoding can facilitate species identification in the *R. canescens* complex [9]. However, the taxon sampling of these molecular studies was too limited to infer species delimitations within the *R. canescens* complex with confidence.

In the present study, molecular species delimitations and relationships in the *R. canescens* complex are assessed based on accessions from all species of the complex, including *R. varium*. According to previous analyses [9], [14], [15], the plastid (cpDNA) *rps4-trnT-trnL* and nuclear ribosomal ITS regions were chosen as most promising markers in terms of potential sequence variability and sequencing success. We aim to infer whether (i) the morphological taxa of the *R. canescens* complex can be distinguished at the molecular level and whether the degree of genetic differentiation supports their recognition at the species level, (ii) morphological characters used for species identification are suitable in the light of the molecular data, and (iii) intraspecific molecular diversity corresponds to hitherto recognized intraspecific taxa. We will discuss which part of the sequenced DNA regions is most suitable for molecular species identification and the implications thereof for DNA barcoding of mosses, in particular in complexes of closely related species.

## Results

The *rps4-trnT-trnL* region could be amplified and sequenced from 68 out of the 70 newly analyzed *Racomitrium* specimens. From two specimens only the *rps4-trnT* part could be amplified. The complete ITS region (ITS1-5.8S-ITS2) was obtained from all 70 specimens, except that in one specimen the 5′ end of ITS1 remained incomplete. Considering also the six additional specimens which could not or only partially be amplified and were excluded from further analyses, amplification and sequencing success was 90% for the complete *rps4-trnT-trnL* region and 92% for ITS.

The alignment of the *rps4-trnT-trnL* region comprised 992 positions and included the 3′ end of the *rps4* gene (positions 1–124), *rps4*-*trnT*_UGU_ intergenic spacer (125–462), *trnT*_UGU_ gene (463–536), *trnT*_UGU_-*trnL*_UAA_ spacer (537–870), *trnL*_UAA_ 5′ exon (871–905), and the 5′ part of the *trnL*_UAA_ intron (906–992). The nrITS alignment comprised 1418 positions and included the internal transcribed spacer 1 (positions 1–817), 5.8S rRNA gene (818–975), internal transcribed spacer 2 (976–1409), and the 5′end of the 26S rRNA gene (1410–1418). Parsimony-informative positions were 48 in the plastid region (26 substitutions/22 indels coded by simple indel coding [SIC]) and 308 (134/174) in ITS, resulting in a total of 356 parsimony-informative positions including indels.

**Figure 1:**
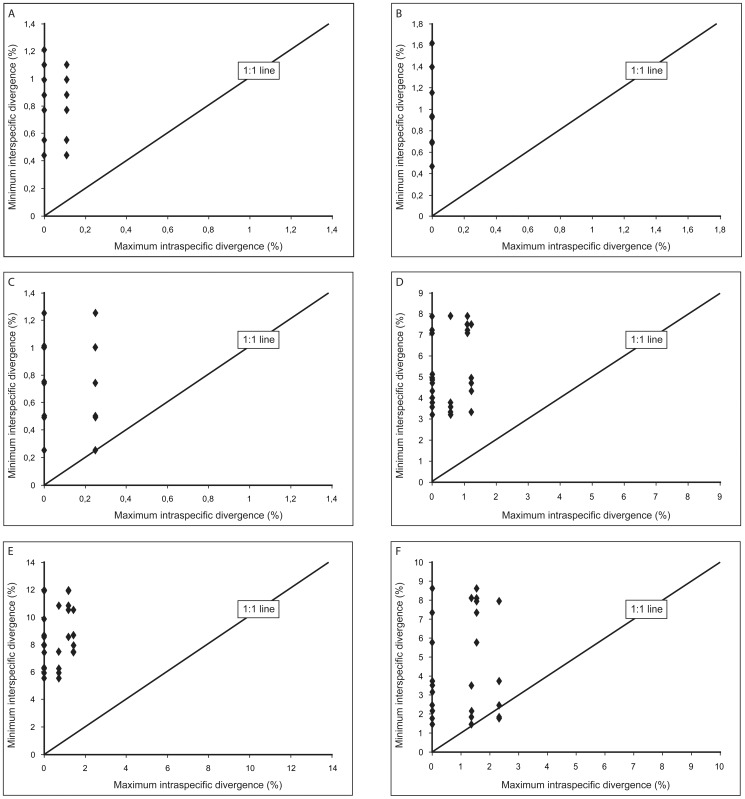
Sequence divergence percentages between species pairs in the *Racomitrium canescens* complex. Comparison of maximum intraspecific versus minimum interspecific divergence percentages for species pairs of five species (*R. canescens*, *R. elongatum*, *R. ericoides*, *R. japonicum*, *R. muticum*) with more than one specimen sequenced for the plastid *rps4-trnT-trnL* region (A) and its partitions *rps4-trnT* (B) and *trnT-trnL* (C) as well as the nrITS region (D) and its partitions ITS1 (E) and ITS2 (F).

Ranges of intraspecific versus interspecific pairwise nucleotide distances according to the Kimura 2-parameter (K2P) model overlapped in the combined dataset and the ITS partitions (Table 1). This was due to small interspecific distances between *R. muticum* and *R. pygmaeum* on the one hand and rather large intraspecific distances within *R. japonicum* on the other. To further compare the different partitions, maximum intraspecific divergences were plotted against minimum interspecific divergences for all possible species pairs of the five species with more than one specimen sequenced (*R. canescens*, *R. elongatum*, *R. ericoides*, *R. japonicum*, *R. muticum*). The resulting graphs (Fig. 1A–F) showed that interspecific divergences were clearly greater than intraspecific variation except for few pairwise comparisons in *trnT-trnL* and ITS2, i.e., that a barcoding gap was present (data points above the 1∶1 line). Tables of all nucleotide distances measured are available on request. Significant p-values were obtained for all pairwise comparisons of Fst estimates based on plastid and ITS haplotypes for the the five species with more than one specimen sequenced (Table 2), except for ITS of *R. japonicum*–*R. panschii*.

**Table 1:** Intra- versus interspecific pairwise distances of *rps4-trnT-trnL* and ITS sequences in the *Racomitrium canescens* complex.

	combined	*rps4-trnT-trnL*	*rps4-trnT*	*trnT-trnL*	ITS	ITS1	ITS2
intra	0–0.0068	0–0.0011	0	0–0.0025	0–0.0135	0–0.0173	0–0.0223
inter	0.0056–0.0511	0.0022–0.0132	0.0023–0.0161	0.0025–0.0151	0.0094–0.0913	0.0149–0.1686	0.0069–0.1006
overlap	0.0012	0	0	0	0.0041	0.0024	0.0154

Kimura 2-parameter (K2P) distances are shown for the combined molecular markers and different partitions thereof. The upper two rows indicate the ranges of intraspecific and interspecific distances for all eight species of the *R. canescens* complex plus *R. varium*. The last row indicates the overlap between the maximum intraspecific and minimum interspecific distances.

**Table 2:** Calculations of pairwise Fst estimates and p-values for five species of the *Racomitrium canescens* complex.

	*R. japonicum*	*R. elongatum*	*R. panschii*	*R. ericoides*	*R. canescens*
*R. japonicum*		0.907/0.001*	0.253/0.096*	0.286/0.039*	0.253/0.078*
*R. elongatum*	0.465/0.002*		0.796/0.000*	0.619/0.000*	0.549/0.000*
*R. panschii*	1.000/0.166*	0.515/0.000*		0.243/0.002*	0.214/0.006*
*R. ericoides*	1.000/0.001*	0.661/0.000*	1.000/0.000*		0.244/0.000*
*R. canescens*	0.502/0.000*	0.335/0.000*	0.537/0.000*	0.641/0.000*	

Group comparisons based on the ITS sequence and indel data are indicated above the diagonal, whereas group comparisons based on the plastid sequence and indel data are shown below the diagonal. Significant p-values (significance level 0.05) are indicated by an asterisk.

Separate phylogenetic reconstructions under maximum parsimony (MP) of the *rps4-trnT-trnL* versus ITS sequences and of the four smaller partitions (*rps4-trnT*, *trnT-trnL*, ITS1, and ITS2) resulted in differently resolved trees, but did not show incongruence with respect to significantly supported clades (Supporting Figs. S1, S2, S3, S4, S5, S6). The partition homogeneity test (ILD test) between the ITS and plastid alignments did not indicate the presence of incongruence (*P* = 0.8). Bootstrap support values of the clades of the *Racomitrium canescens* complex, including *R. varium*, which were obtained in the separate analyses, are compared in Table 3.

**Table 3:** Comparison of maximum parsimony bootstrap support (in %) for clades of species of the *Racomitrium canescens* complex.

	*rps4-trnT-trnL*	*rps4-trnT*	*trnT-trnL*	ITS	ITS1	ITS2
*R. canescens*	**94**/**88**	64/–	**88**/**88**	**100**/**100**	**100**/**100**	63/**85**
*R. elongatum*	**85**/**90**	62/**73**	62/66	**100**/**100**	**100**/**100**	**81**/**100**
*R. ericoides*	**93**/**99**	**83**/**91**	63/**89**	**96**/**100**	**92**/**100**	59/**79**
*R. panschii*	**87**/**95**	**88**/**86**	–/**84**	**99**/**100**	**99**/**99**	**84**/**99**
*R. varium*+(*R. barbuloides*+*R. japonicum*)	–/–	–/–	–/–	**95/85**	–/–	**99/92**
*R. barbuloides*+*R. japonicum*	**86**/**100**	**84**/**100**	–/–	**100**/**100**	**100**/**100**	**94**/**100**
*R. japonicum*	**77**/**81**	63/68	**74**/**92**	**100**/**100**	**100**/**100**	**100**/**100**
*R. pygmaeum*+*R. muticum*	63/54	–/–	64/–	**100**/**100**	**97**/**100**	56/64
*R. muticum*	–/–	–/–	–/–	**95**/**100**	**94**/**100**	–/56

The bootstrap analyses were performed using parts of the analyzed DNA regions *rps4-trnT-trnL* and nrITS, which can be amplified separately with established primers for species identification purposes (DNA barcoding). Two specimens with missing sequences were excluded from the analysis of *trnT-trnL*. Values before and after the dash are from analyses without and with indels included by simple indel coding (SIC), respectively; values >70% are in bold. Dashes denote clades that were not resolved in the respective phylogenetic reconstructions.

**Figure 2:**
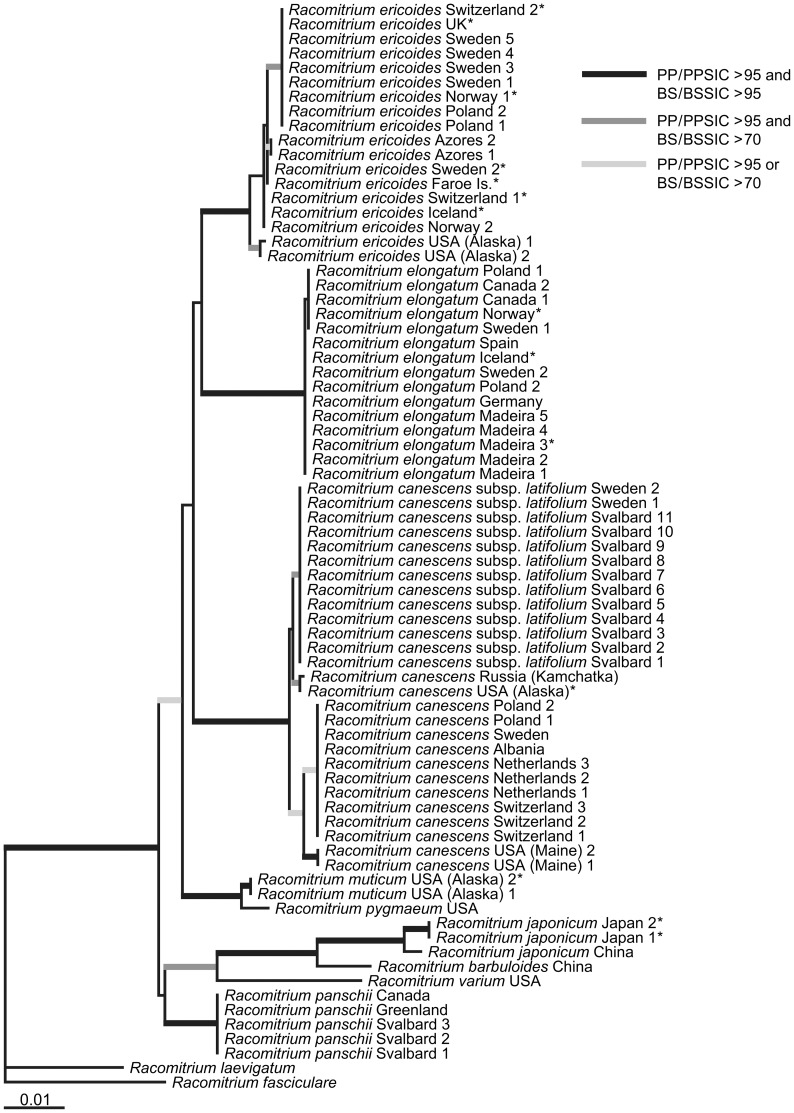
Molecular species circumscriptions and relationships in the *Racomitrium canescens* species complex. Single optimal maximum likelihood tree of 73 specimens based on combined plastid *rps4-trnT-trnL* and nrITS sequences. *Racomitrium fasciculare* and *R. laevigatum* were used as outgroup representatives. Thick lines indicate bootstrap support (BS) values from respective maximum parsimony and significant posterior probabilities (PP) from respective Bayesian analysis: BS>95% and PP>95 (black), BS>70% and PP>95 (dark grey), BS>70% or PP>95 (light grey). Asterisks indicate specimens whose identification was revised according to their position in the molecular phylogenetic reconstructions.

In the MP-PRAP analyses of the combined dataset, 525 trees (lengths 351 steps, consistency index CI = 0.815, retention index RI = 0.964) were retained without indels and 1113 trees (lengths 750, CI = 0.700, RI = 0.953) with indels coded by SIC included. A maximum likelihood calculation recovered a single optimal tree (ln*L* = −5429.03137), which is depicted in Fig. 2, with statistical support from MP analyses (bootstrap support values, BS) and Bayesian inference (BI; posterior probability values, PP), both without and with indels, indicated. As shown in Fig. 2, the *Racomitrium canescens* complex, including *R. varium*, is well supported in all MP and BI analyses. Within the complex, all species with more than one specimen included (*R. canescens*, *R. elongatum*, *R. ericoides*, *R. japonicum*, and *R. muticum*) form clades with significant statistical support. The relationships of *R. barbuloides* as sister to *R. japonicum*, *R. varium* as sister to these two species, and *R. pygmaeum* as sister to *R. muticum* receive significant support as well. Relationships between these clades and the other species, i.e., the backbone of the phylogenetic reconstruction, however, remain unsupported or receive significant support in the Bayesian analyses only. Intraspecific variation is observed in *R. canescens* s.str., *R. ericoides*, and *R. japonicum*, whereas sequences are almost or even completely identical in *R. elongatum* and *R. panschii*. One clade within *R. canescens* s.str. corresponds to *R. canescens* subsp. *latifolium*.

## Discussion

### Species circumscriptions and relationships in the *Racomitrium canescens* complex

All eight morphologically defined species of the *R. canescens* complex plus *R. varium* are easily distinguishable from each other based on the combined *rps4-trnT-trnL* and ITS sequence data (Fig. 2). Much confusion about species delimitations in the *R. canescens* complex arose from early taxonomic attempts to subdivide *R. canescens* s.l. into various varieties or forms (overview in [17]), which were often based on environmental modifications of morphological characters only. Frisvoll [17] emphasized the frequent presence of mixed populations (‘mixed stands’) of morphologically distinguishable plants, which indicated that a number of genetically distinct taxa do exist in the *R. canescens* complex. In fact, the species formerly treated as varieties of *R. canescens*, namely *R. barbuloides* (*R. canescens* var. *epilosum* H. Müll. ex Milde, fide [20]), *R. ericoides*, and *R. muticum* [17], [18], and the species newly described by Frisvoll [17], viz. *R. elongatum* and *R. pygmaeum*, are molecularly clearly separated from *R. canescens* s.str.

Despite low phylogenetic distances in the *R. canescens* complex, genetic differentiation between species is generally larger than within species, as inferred from the generally smaller intraspecific than interspecific distances (Fig. 1, branch lengths in Fig. 2) and the significant pairwise Fst comparisons for five species of the complex (Table 2). It has to be noted, however, that the Fst estimates are based on a small sampling and only a small part of the genomes with few haplotypes. Furthermore, no incongruence between the plastid and ITS sequences was found, rejecting the occurrence of hybridization in the present dataset. The overlap in the total sequence distance ranges (Table 1) is due to an exception of the general observation of smaller intraspecific than interspecific distances, as the molecular divergence in ITS between *R. muticum* and *R. pygmaeum* is smaller than the intraspecific divergence within *R. japonicum*. It could therefore be argued that the former two should be treated as one species, whereas *R. japonicum* should be split into two. However, *R. muticum* and *R. pygmaeum* are easily distinguishable by morphological characters [15]–[18]. *Racomitrium japonicum*, in contrast, shows only little morphological variation, and if split, distinguishing the two segregate taxa might be only possible by molecular characters and possibly a geographic separation (China versus Japan, cf. Fig. 2). This needs to be tested by analyzing a larger number of specimens from the entire East Asian distribution area of *R. japonicum*.

Based on the present inferences we argue that the molecular data support Frisvoll's [17] thorough revision and that his morphological species circumscriptions should be maintained, as they correspond to well-supported clades in the molecular phylogenetic reconstructions. Molecular species delimitation in the *R. canescens* complex is thus straightforward, in contrast to several other genera of liverworts and mosses analyzed recently, where the presence of para- or polyphyletic species, cryptic speciation, incongruence between molecular markers, or incongruence between molecular and morphological characters complicated species delimitation (e.g. [2], [4], [21]–[24]).

In line with distinguishing the taxa of the *R. canescens* complex at the species level, intraspecific molecular divergence could be treated taxonomically at the subspecies level, especially when groups of specimens form at least moderately supported subclades, such as within *R. canescens*, *R. ericoides*, and *R. japonicum* (Fig. 2). In fact, one of the subclades within *R. canescens* corresponds to *R. canescens* subsp. *latifolium* (Fig. 2), which is morphologically and geographically separated from *R. canescens* subsp. *canescens*, although both morphological characters and the distribution range overlap between the two subspecies [17]. The taxonomic status of other intraspecific clades that are molecularly distinguishable within the widespread *Racomitrium* species remains to be tested. As in *R. japonicum*, subclades in *R. canescens* seem to be geographically separated (circum-North Pacific, western North America, Europe), which would provide support for treating them as separate subspecies in addition to subsp. *latifolium*, but more specimens, especially from outside Europe, need to be analyzed.

At the supraspecific level, the present molecular data do not support the division of the *R. canescens* complex into two groups of species, which were formerly treated as *Racomitrium* subsections *Canescens* and *Ericoides* [17] or *Niphotrichum* sections *Niphotrichum* and *Elongata* [18], respectively. According to these classifications, *R. canescens* s.str. and *R. panschii* should be closely related (both placed in subsect. *Canescens*) and separated from the remaining species of the complex (classified in subsect. *Ericoides*). However, the present molecular data indicate a closer relationship of *R. panschii* with *R. barbuloides*, *R. japonicum*, and *R. varium* (Fig. 2). *Racomitrium japonicum*, which was considered by Frisvoll [17] as distantly related to the remaining taxa of the *R. canescens* complex, is found here closely related to *R. barbuloides* (Fig. 2), with which it frequently grows together in mixed stands [17]. Both *R. barbuloides* and *R. japonicum* are predominantly East Asian species, the latter reaching southwards to Vietnam and Australia [25], [26]. Similarly, a close relationship is indicated between the circum-North Pacific *R. muticum*, which is most frequent in western North America [18], and the western North American endemic *R. pygmaeum*. Further inferences on species relationships as well as analyses of phylogeographic patterns in the *R. canescens* complex need further study based on a denser sampling especially in the North American-East Asian region and sequencing of additional markers to increase support for the backbone of the phylogenetic reconstruction (Fig. 2).

**Figure 3:**
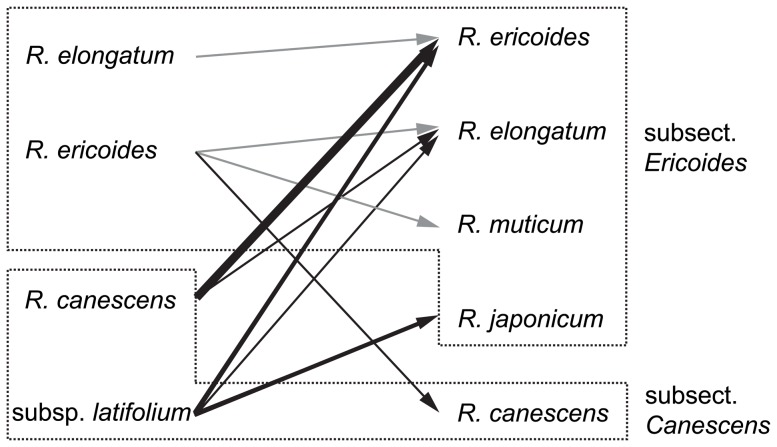
Revised species identifications in the *Racomitrium canescens* species complex based on molecular data. Changes in species identification of 14 specimens analyzed in the present study are indicated by arrows. Arrow thickness is equivalent to the number of specimens transferred from one species to another (one, two, or four specimens, respectively). Grey or black arrows indicate changes within or between subsections *Canescens* and *Ericoides* [15], respectively.

The clear molecular distinction between the species of the *R. canescens* complex indicates that the morphological characters used to identify them (leaf shape, hairpoint structure and stance, nerve length, alar cells and basal marginal leaf cells), albeit being in part rather small and difficult to apply, are reliable for species identification. On the other hand, misidentification of collections seems to be a severe problem in the *R. canescens* complex. If the molecular phylogenetic reconstructions accurately reflect species delimitations, 14 out of the 70 newly sequenced specimens (20%) were misidentified based on morphological characters (Figs. 2, 3). This could partly be due to mixed collections or field determinations without later checking the material microscopically (e.g. collections provisionally named *R. canescens* [*sensu lato*] to indicate that they belong to the complex), but might also be an inherent problem of the diagnostic characters. The majority of the 14 misidentified specimens belonged to species of subsection *Ericoides* sensu Frisvoll [17], not to *R. canescens* s.str. or subsp. *latifolium* (Fig. 3). Morphological re-investigation revealed that some of the collections named *R. canescens*, but actually belonging to *R. ericoides* or *R. elongatum*, were intermediate in terms of the diagnostic characters separating these three species. They showed rather broad and obtusely keeled leaves, typical for *R. canescens*, but a single, non-forked nerve reaching at least ¾ up the leaf, typical for the two latter species, members of subsect. *Ericoides*. The suitability of leaf shape as a diagnostic character hence is questionable, but this needs to be confirmed by analysis of a larger number of collections labelled *R. canescens*.

### Implications for DNA barcoding of species complexes in mosses

Species identification by DNA barcoding seems to be more difficult in bryophytes than in other land plant lineages or in animals. Instead of using a single, short piece of DNA to discriminate species across a wide range of lineages, barcoding in land plants is supposed to be based on one or two core markers, plus additional information from other DNA regions where necessary (e.g. [11], [27], [28]). Different marker combinations, mostly from the plastid genome, have been proposed (see [11] for review), such as *rbcL* and *matK* as core markers for land plants [5], or the *psbA-trnH* spacer together with the nrITS region for angiosperms [29]. In bryophytes, especially mosses, however, the respective plastid markers either tend to be short (*psbA-trnH* spacer, *trnL-F* spacer, [30], [31]), their discrimination capacity at the species level is still debated (*rbcL*, [8], [10]), or efforts are still needed concerning primer design and amplification strategy (*trnK*/*matK*, e.g. [7]). A stand-alone barcoding marker is unlikely to be found among the standard plastid markers used for phylogeny reconstruction [11]. Liu et al. [8] identified five plastid markers with amplification success >90% and taxon assignment success >80% (in different combinations up to 92%), viz. *rbcL*, *rpoC1*, *rps4*, *psbA- trnH*, and *trnL-F*. The suitability of these markers for discriminating closely related moss species in species complexes, however, remained difficult to assess, as only few species from selected genera were compared. The subsequent study focusing on Grimmiaceae [9] already showed that (partial) *rbcL* sequences did not perform well, and the discrimination capacity of the best marker (*psbA- trnH*) did not exceed 65% in the whole family. In *Racomitrium*, all 11 species included in Liu et al. [9] could be distinguished by *rps4*, and with >80% success by *rbcL* and *psbA-trnH*, but again the question arises whether these markers would provide sufficient information when further closely related species are compared.

In the present study of the *Racomitrium canescens* complex, almost no differences were observed between the plastid *rps4-trnT-trnL* and nuclear ribosomal ITS regions concerning amplification and sequencing success. Both regions could be sequenced completely for at least 90% of the analyzed specimens. The ITS region showed a clear gap between intra- and interspecific sequence divergence for the species represented by more than one accession (Fig. 1) and allowed to resolve clades of all species of the complex with significant statistical support (Table 3). The same was true for ITS1 alone (the clade of *R. varium*, *R. barbuloides* and *R. japonicum* was not resolved, but *R. varium* still formed a clade separate from the other species). Although the different parts of the *rps4-trnT-trnL* region and the ITS2 performed well, too (Fig. 1), they failed to discriminate *R. pygmaeum* and *R. muticum* (Table 3). Including indels by simple indel coding did not significantly improve discrimination capacity of any marker and in some cases even resulted in lower clade support (Table 3). Here a different indel coding strategy might be desired.

Advantages and disadvantages of using the nrITS region as DNA barcode have been discussed, e.g., by [29], [32]. Apart from inherent problems such as the possible presence of paralogous ITS copies or incongruence between ITS1 and ITS2 (e.g. [33]), amplification difficulties of the whole ITS1-5.8S-ITS2 region need to be taken into account. The latter, however, can often be solved by amplifying ITS1 and ITS2 separately, which has also been done in phylogenetic analyses of bryophytes (e.g. [34]). Until now, mostly ITS2 has been considered as plant DNA barcode marker [32], [35]. Bell et al. [7] considered the whole ITS region for the liverwort genus *Herbertus*. However, amplification and sequencing success was lower than in the employed plastid markers. With respect to the results of the present study, we argue that ITS1, which is generally more variable than ITS2 [36], should also be considered as potential barcode marker in complexes of closely related species of mosses. The preliminary observation in the *Racomitrium canescens* complex that ITS1 seems to outperform ITS2 should be tested in further species complexes to decide which part of the nrITS region works best as potential core barcoding marker for species identification in mosses. Furthermore, DNA barcoding in difficult species complexes should be complemented by redefining morphological characters, to develop a set of easy-to-use molecular and non-molecular identification tools for improving biodiversity assessments and ecological research, including mosses.

## Materials and Methods

### Ethics statement

All necessary permits were obtained for field studies to collect material used for molecular analysis in Svalbard (Governor of Svalbard, references 2008/00688-2, 2009/00412), Greenland (BioBasis project, Dr. N.M. Schmidt, Aarhus University), Madeira (Dr. S. Fontinha, National Park of Madeira Services/Madeira University), Azores (Secretaria Regional da Agricultura e Florestas Dos Açores), UK (H. Cole, Manager/Senior Ranger Naturalist, Ben Lawers National Nature Reserve), and The Netherlands (Dr. H. van der Hagen, Dunea N.V.). Further material was collected in areas without specific permissions required or concerned herbarium collections from G, KRAM, L, and S, which were used according to regulations of the respective herbaria. Areas without permission needed were neither privately owned nor protected. The species sampled are neither protected nor listed by CITES (Convention on the International Trade in Endangered Species).

### Taxon sampling, DNA extraction, PCR and sequencing

The present taxon sampling and outgroup selection was based on recent molecular phylogenetic reconstructions of *Racomitrium* s.l., which showed that *R. fasciculare* and *R. laevigatum* (both treated as *Codriophorus* by [13]), formed a sister clade to the *R. canescens* complex [15], [19] (treated as *Niphotrichum* by [13]), the latter including *R. varium* [15] (also treated as a *Codriophorus* in [13]). Sequences from the plastid *rps4-trnT-trnL* and nuclear ribosomal ITS regions were newly generated for 70 specimens of the *Racomitrium canescens* complex. Voucher information and Genbank accession numbers are listed in Appendix S1. Six further collections could not or only partially be sequenced or the sequences were of insufficient quality. In addition, sequences of five *Racomitrium* specimens were taken from earlier studies, namely *R. elongatum* (Spain) from [14] and *R. ericoides* (both samples from Poland) as well as *R. fasciculare* and *R. laevigatum* as outgroup representatives from [19].

Distal parts of single shoots were thoroughly cleaned with distilled water. Total genomic DNA was extracted using the DNeasy® Plant Kit (Qiagen) or the NucleoSpin® Plant II Kit (Macherey-Nagel). The employed molecular markers were amplified by PCR using protocols and primers as described in [14] for *rps4-trnT-trnL* (primers rps4-166F and P6/7) and [37] for nrITS (primers 18F and 25R). In a few cases of difficulties with obtaining PCR products, the *rps4-trnL* part was split into two halves, which were amplified and sequenced separately with primers rps4-166F and A-Rbryo or A-Fbryo and P6/7, respectively [14]. PCR products were purified using the Wizard® DNA Clean-up kit (Promega) or by Macrogen Inc. (www.macrogen.com), where the automated sequencing was performed as well. Sequencing primers were those used for PCR.

### Alignment, sequence analysis and phylogenetic reconstructions

DNA sequences were manually aligned in PhyDE® v0.995 [38]. Phylogenetic reconstructions of taxon circumscriptions and relationships were performed based on the maximum parsimony (MP) principle and using two model-based approaches, maximum likelihood (ML) and Bayesian inference (BI). Separate MP analyses of the *rps4-trnT-trnL* versus ITS sequences were performed and the resulting tree topologies were checked for possible incongruence between the plastid and nuclear markers by visual inspection and by applying a partition homogeneity test (ILD test) [39], [40] as implemented in PAUP* 4.0b10 [41] (100 replicates). Calculations of pairwise Fst estimates and p-values based on plastid and ITS haplotypes were performed using Arlequin v3.5.1.3 [42], with haplotypes (including indel characters) delineated in TCS v1.2.1 [43].

Phylogenetic analyses of the combined plastid and nuclear markers were run using MP, ML, and BI. To evaluate the employed markers for DNA barcoding, further MP analyses were performed of four partitions for which primers pairs are available, viz. *rps4-trnT*, *trnT-trnL*, ITS1, and ITS2. In addition, pairwise nucleotide distances between all sequences were calculated according to the K2P model (cf. [8], [9]) for the combined dataset and all partitions, and compared between and within the species of the *R. canescens* complex.

Calculation of pairwise distances as well as MP and ML analyses were performed in PAUP. Heuristic searches under parsimony were implemented using random sequence addition with 1000 replicates and tree bisection-reconnection (TBR) branch swapping. All MP analyses were run with gaps (indels) either treated as missing data or coded as informative by a simple indel coding (SIC) strategy [44] as implemented in SeqState [45]. Heuristic bootstrap searches under parsimony were performed with 1000 replicates and 10 random addition cycles per bootstrap replicate with the same options in effect. To search the tree space for islands of more parsimonious trees, parsimony ratchet analyses were performed with PRAP2 [46] in combination with PAUP, employing the default options (200 iterations, 25% of randomly chosen positions up-weighted to 2) and superimposed 10 random addition cycles.

For the model-based approaches, model testing was performed in Modeltest 3.7 [47] employing MrMTgui [48]. GTR+Γ+I was indicated as best-fit model of the combined dataset according to the Akaike information criterion (AIC). Consequently, the settings basefreq = (0.3011 0.2002 0.2254), nst = 6, Rmat = (1.5235 5.1208 0.6544 2.3313 7.1000), rates = gamma, shape = 0.7849, and pinvar = 0.6263 were used for ML, and nst = 6 and rates = invgamma for BI. Bayesian posterior probabilities were calculated based on the Metropolis-coupled Markov chain Monte Carlo (MCMCMC) method, using MrBayes v3.1 [49]. In a second set of Bayesian analyses the indels coded by SIC were included, with sequence and indel data treated as separate and unlinked partitions, employing the restriction site model (‘F81’) for the indel matrix. The a priori probabilities supplied were those specified in the default settings of the program. Four runs with four chains (10^6^ generations each) were run simultaneously, with the temperature of the single heated chain set to 0.2. Chains were sampled every 1000 generations and the respective trees written to a tree file. Fifty percent majority rule consensus trees and posterior probabilities of clades were calculated by combining the four runs and using the trees sampled after the chains converged. Trace plots generated in Tracer v1.5 [50] were used to check for convergence of the runs (plateaus of all runs at comparable likelihoods) and to infer the ‘burnin’, which ranged approximately between the first 100000 and 120000 generations (first 100–120 sampled trees). Consequently, the first 150 trees (15%) were deleted to be sure that only trees of the stationary phase were included.

## Supporting Information

Figure S1 **Maximum parsimony phylogenetic reconstruction of the** ***Racomitrium canescens*** **species complex based on plastid** ***rps4-trnT-trnL*** **sequences.** Indels coded by simple indel coding were included. Bootstrap support values of the respective analyses without indels (before the slash) and with indels (after the slash) are indicated.
(TIF)

Figure S2 **Maximum parsimony phylogenetic reconstruction of the** ***Racomitrium canescens*** **species complex based on plastid** ***rps4-trnT*** **sequences.** Indels coded by simple indel coding were included. Bootstrap support values of the respective analyses without indels (before the slash) and with indels (after the slash) are indicated.
(TIF)

Figure S3 **Maximum parsimony phylogenetic reconstruction of the** ***Racomitrium canescens*** **species complex based on plastid** ***trnT-trnL*** **sequences.** Indels coded by simple indel coding were included. Bootstrap support values of the respective analyses without indels (before the slash) and with indels (after the slash) are indicated.
(TIF)

Figure S4 **Maximum parsimony phylogenetic reconstruction of the** ***Racomitrium canescens*** **species complex based on nuclear ribosomal ITS sequences.** Indels coded by simple indel coding were included. Bootstrap support values of the respective analyses without indels (before the slash) and with indels (after the slash) are indicated.
(TIF)

Figure S5 **Maximum parsimony phylogenetic reconstruction of the** ***Racomitrium canescens*** **species complex based on nuclear ribosomal ITS1 sequences.** Indels coded by simple indel coding were included. Bootstrap support values of the respective analyses without indels (before the slash) and with indels (after the slash) are indicated.
(TIF)

Figure S6 **Maximum parsimony phylogenetic reconstruction of the** ***Racomitrium canescens*** **species complex based on nuclear ribosomal ITS2 sequences.** Indels coded by simple indel coding were included. Bootstrap support values of the respective analyses without indels (before the slash) and with indels (after the slash) are indicated.
(TIF)

Appendix S1 **Geographic origin (with numbering corresponding to** **Fig. 1** **of the manuscript), voucher information and herbarium locations (in brackets), and GenBank accession numbers (** ***rps4-trnT-trnL*** **, nrITS) of 70** ***Racomitrium*** **specimens newly sequenced for the present study.**
(DOC)

## References

[1] Shaw AJ (2009) Bryophyte species and speciation. In: Goffinet B, Shaw AJ, editors. Bryophyte Biology, 2^nd^ ed. Cambridge: Cambridge University Press.

[2] Vanderpoorten A, Shaw AJ (2010) The application of molecular data to the phylogenetic delimitation of species in bryophytes: A note of caution. Phytotaxa 9: 229–237.

[3] Shaw AJ (2001) Biogeographic patterns and cryptic speciation in bryophytes. J Biogeogr 28: 253–261.

[4] Heinrichs J, Klugmann F, Hentschel J, Schneider H (2009) DNA taxonomy, cryptic speciation and diversification of the Neotropical-African liverwort, *Marchesinia brachiata* (Lejeuneaceae, Porellales). Mol Phylogenet Evol 53: 113–121. 19501177 10.1016/j.ympev.2009.05.032

[5] CBOL Plant Working Group (2009) A DNA barcode for land plants. Proc Natl Acad Sci USA 106: 12794–12797. 19666622 10.1073/pnas.0905845106 PMC2722355

[6] Korotkova N, Borsch T, Quandt D, Taylor N, Müller K, et al (2011) Epiphytic cacti (Rhipsalideae): How much does it take to resolve relationships and to identify species with molecular markers? Am J Bot 98: 1549–1572. 21900612 10.3732/ajb.1000502

[7] Bell D, Long DG, Forrest AD, Hollingsworth ML, Blom HH, et al (2011) DNA barcoding of European *Herbertus* (Marchantiopsida, Herbertaceae) and the discovery and description of a new species. Mol Ecol Resources 12: 36–47. 10.1111/j.1755-0998.2011.03053.x 21824334

[8] Liu Y, Yan H-F, Cao T, Ge X-J (2010) Evaluation of 10 plant barcodes in Bryophyta (Mosses). J Syst Evol 48: 36–46.

[9] Liu Y, Cao T, Ge X-Y (2011) A case study of DNA barcoding in Chinese Grimmiaceae and a moss recorded in China for the first time. Taxon 60: 185–193.

[10] Stech M, Quandt D (2010) 20,000 species and five key markers: the status of molecular bryophyte phylogenetics. Phytotaxa 9: 196–228.

[11] Hollingsworth PM, Graham SW, Little DP (2011) Choosing and using a plant DNA barcode. PloS ONE 6: e19254. 21637336 10.1371/journal.pone.0019254 PMC3102656

[12] Stenøien HK (2008) Slow molecular evolution in 18S rDNA, *rbc*L and *nad*5 genes of mosses compared with higher plants. J Evol Biol 21: 566–571. 18205784 10.1111/j.1420-9101.2007.01479.x

[13] Ochyra R, Żarnowiec J, Bednarek-Ochyra H (2003) Census catalogue of Polish mosses. Biodiv Poland 3: 1–372.

[14] Hernández-Maqueda R, Quandt D, Werner O, Muñoz J (2008) Phylogeny and classification of the Grimmiaceae/Ptychomitriaceae complex (Bryophyta) inferred from cpDNA. Mol Phylogenet Evol 46: 863–877. 18262799 10.1016/j.ympev.2007.12.017

[15] Larraín J (2011) Phylogeny of the genus *Racomitrium* (Bryophyta, Grimmiaceae) and taxonomy of the Latin American species. PhD thesis, University of Concepción, Chile.

[16] Heinonen K (1971) *Racomitrium canescens* (Hedw.) Brid. and *R. ericoides* (Hedw.) Brid. (Bryophyta, Grimmiaceae) in northwestern Europe. Ann Bot Fennici 8: 142–151.

[17] Frisvoll AA (1983) A taxonomic revision of the *Racomitrium canescens* group (Bryophyta, Grimmiales). Gunneria 41: 1–181.

[18] Ochyra R, Bednarek-Ochyra H (2007) Grimmiaceae. 7. *Niphotrichum* . Flora of North America 27: 285–292.

[19] Larraín J, Quandt D, Muñoz J (2011) *Bucklandiella araucana* (Grimmiaceae), a new species from Chile. Bryologist 114: 732–743.

[20] Noguchi A (1974) Musci Japonici. X. The genus *Racomitrium* . J Hattori Bot Lab 38: 337–369.

[21] Stech M, Wagner D (2005) Molecular relationships, biogeography, and evolution of Gondwanan *Campylopus* species (Dicranaceae, Bryopsida). Taxon 54: 377–382.

[22] Huttunen S, Ignatov MS (2010) Evolution and taxonomy of aquatic species in the genus *Rhynchostegium* (Brachytheciaceae, Bryophyta). Taxon 59: 791–808.

[23] Hedenäs L (2011) Incongruence among morphological species circumscriptions and two molecular datasets in *Sarmentypnum* (Bryophyta: Calliergonaceae). Taxon 60: 1596–1606.

[24] Sukkharak P, Gradstein SR, Stech M (2011) Phylogeny, taxon circumscriptions and character evolution in the core Ptychanthoideae (Lejeuneaceae, Marchantiophyta). Taxon 60: 1607–1622.

[25] Vitt DH, Cao T, Frisvoll AA (1993) *Racomitrium leptostomoides* and *R. szuchuanicum*, new synonyms of *R. japonicum* Dozy & Molk. (Bryopsida). Nova Hedwigia 57: 457–461.

[26] Gao C, Crosby MR, He S (2003) Moss Flora of China, Vol. 3. Grimmiaceae–Tetraphidaceae. Beijing, New York & St. Louis: Science Press & Missouri Botanical Garden.

[27] Pennisi E (2007) Taxonomy – Wanted: A barcode for plants. Science 318: 190–191. 17932267 10.1126/science.318.5848.190

[28] Fazekas AJ, Kesanakurti PR, Burgess KS, Percy DM, Graham SW, et al (2009) Are plant species inherently harder to discriminate than animal species using DNA barcoding markers? Mol Ecol Resources 9 Suppl. 1130–139. 10.1111/j.1755-0998.2009.02652.x 21564972

[29] Kress WJ, Wurdack KJ, Zimmer EA, Weigt LA, Janzen DH (2005) Use of DNA barcodes to identify flowering plants. Proc Natl Acad Sci USA 102: 8369–8374. 15928076 10.1073/pnas.0503123102 PMC1142120

[30] Quandt D, Stech M (2005) Molecular evolution and secondary structure of the chloroplast *trn*L intron in bryophytes. Mol Phylogenet Evol 36: 429–443. 16005648 10.1016/j.ympev.2005.03.014

[31] Stech M, Frey W (2008) A morpho-molecular classification of the mosses (Bryophyta). Nova Hedwigia 86: 1–21.

[32] Hollingsworth ML, Clark AA, Forrest LL, Richardson J, Pennington RT, et al (2009) Selecting barcoding loci for plants: Evaluation of seven candidate loci with species-level sampling in three divergent gorups of land plants. Mol Ecol Resources 9: 439–457. 10.1111/j.1755-0998.2008.02439.x 21564673

[33] Stech M, Dohrmann J (2004) Molecular relationships and biogeography of two Gondwanan *Campylopus* species, *C. pilifer* and *C. introflexus* (Dicranaceae). Monogr. Syst Bot Missouri Bot Gard 98: 415–431.

[34] Stech M, Werner O, González-Mancebo JM, Patiño J, Sim-Sim M, et al (2011) Phylogenetic inference in *Leucodon* Schwägr. subg. *Leucodon* (Leucodontaceae, Bryophyta) in the North Atlantic region. Taxon 60: 79–88.

[35] Yao H, Song J, Liu C, Luo K, Han J, et al (2010) Use of ITS2 region as the universal DNA barcode for plants and animals. PloS ONE 5: e13102. 20957043 10.1371/journal.pone.0013102 PMC2948509

[36] Vanderpoorten A, Goffinet B, Quandt D (2006) Utility of the internal transcribed spacers of the 18S-5.8S-26S nuclear ribosomal DNA in land plant systematics, with special emphasis on bryophytes. In: Sharma AK, Sharma A, editors. Plant Genome: Biodiversity and Evolution, Vol. 2, Part B. Enfield, New Hampshire: Science Publishers, pp. 385–407.

[37] Stech M, Frahm J-P (1999) The status of *Platyhypnidium mutatum* Ochyra & Vanderpoorten and the systematic value of the Donrichardsiaceae based on molecular data. J Bryol 21: 191–195.

[38] Müller K, Quandt D, Müller J, Neinhuis C (2006) PhyDE®: Phylogenetic Data Editor, version 0.995. Program distributed by the authors. PhyDE website. Available: www.phyde.de. Accessed 2009 Mar 13.

[39] Farris JS, Källersjö M, Kluge AG, Bult C (1994) Testing significance of incongruence. Cladistics 10: 315–319.

[40] Farris JS, Källersjö M, Kluge AG, Bult C (1995) Constructing a significance test for incongruence. Syst Biol 44: 570–572.

[41] Swofford DL (2002) PAUP*: Phylogenetic analysis using parsimony (*and other methods), version 4.0b10. Sunderland, Massachusetts: Sinauer.

[42] Excoffier L, Lischer HEL (2010) Arlequin suite ver 3.5: A new series of programs to perform population genetics analyses under Linux and Windows. Mol Ecol Resources 10: 564–567. 10.1111/j.1755-0998.2010.02847.x 21565059

[43] Clement M, Posada D, Crandall KA (2000) TCS: A computer program to estimate gene genealogies. Mol Ecol 9: 1657–1659. 11050560 10.1046/j.1365-294x.2000.01020.x

[44] Simmons MP, Ochoterena H (2000) Gaps as characters in sequence-based phyogenetic analyses. Syst Biol 49: 369–381. 12118412

[45] Müller K (2004a) SeqState – primer design and sequence statistics for phylogenetic DNA data sets. Appl Bioinformatics 4: 65–69. 10.2165/00822942-200504010-00008 16000015

[46] Müller K (2004b) PRAP – computation of Bremer support for large data sets. Mol Phylogenet Evol 31: 780–782. 15062810 10.1016/j.ympev.2003.12.006

[47] Posada D, Crandall KA (1998) Modeltest: testing the model of DNA substitution. Bioinformatics 14: 817–818. 9918953 10.1093/bioinformatics/14.9.817

[48] Nuin PAS (2005) MTgui—a simple interface to ModelTest. Program distributed by the author. Genedrift website. Available: http://www.genedrift.org/. Accessed 2009 Mar 13.

[49] Huelsenbeck JP, Ronquist F (2001) MrBayes: Bayesian inference of phylogenetic trees. Bioinformatics 17: 754–755. 11524383 10.1093/bioinformatics/17.8.754

[50] Rambaut A, Drummond AJ (2007) Tracer, version 1.5. Program distributed by the authors. University of Edinburgh, Molecular evolution, phylogenetics and epidemiology website. Available: http://tree.bio.ed.ac.uk/software/tracer/. Accessed 2010 Aug 30.

