# Supplementary figures and images for: Molecular Species Delimitation in the Racomitrium canescens Complex (Grimmiaceae) and Implications for DNA Barcoding of Species Complexes in Mosses
Source: PLoS One. 2013 Jan 14;8(1):e53134. doi: 10.1371/journal.pone.0053134 (PMC3544804; doi:10.1371/journal.pone.0053134)

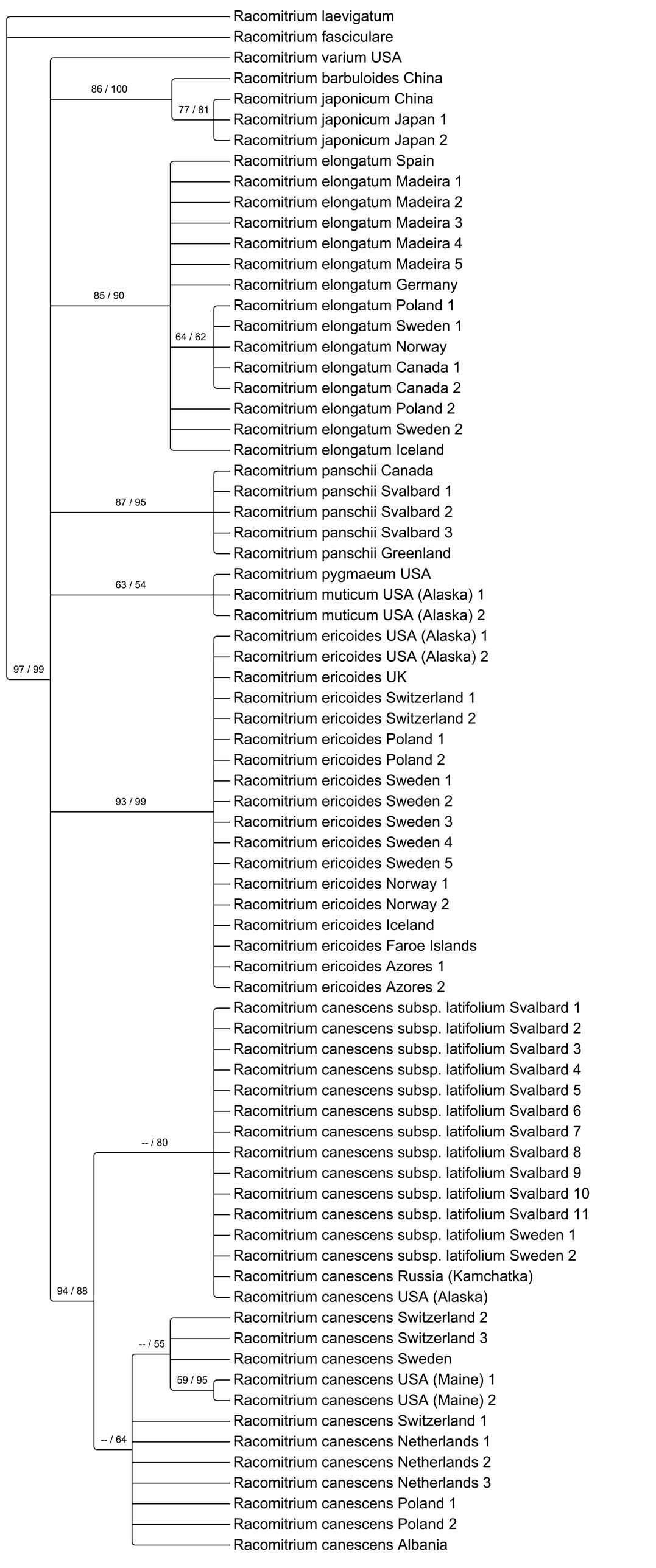

Supplement: Figure S1 — Maximum parsimony phylogenetic reconstruction of the Racomitrium canescens species complex based on plastid rps4-trnT-trnL sequences. Indels coded by simple indel coding were included. Bootstrap support values of the respective analyses without indels (before the slash) and with indels (after the slash) are indicated. (TIF) [file pone.0053134.s001.tif]

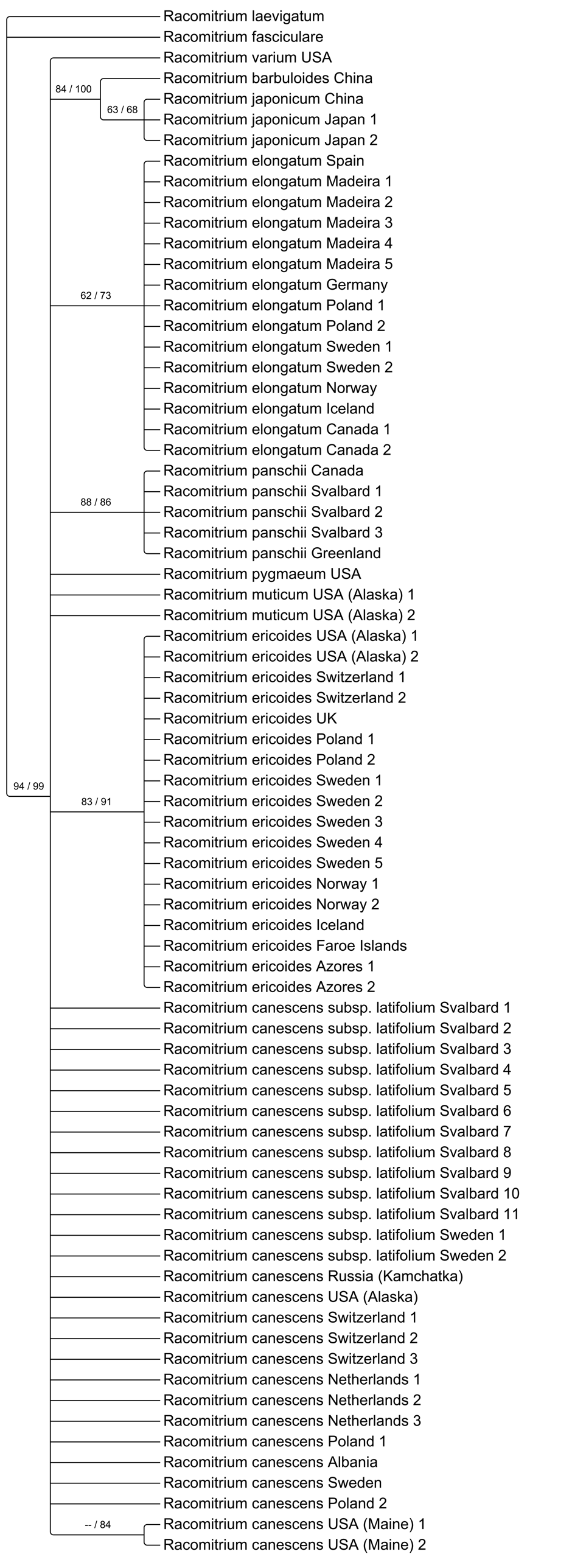

Supplement: Figure S2 — Maximum parsimony phylogenetic reconstruction of the Racomitrium canescens species complex based on plastid rps4-trnT sequences. Indels coded by simple indel coding were included. Bootstrap support values of the respective analyses without indels (before the slash) and with indels (after the slash) are indicated. (TIF) [file pone.0053134.s002.tif]

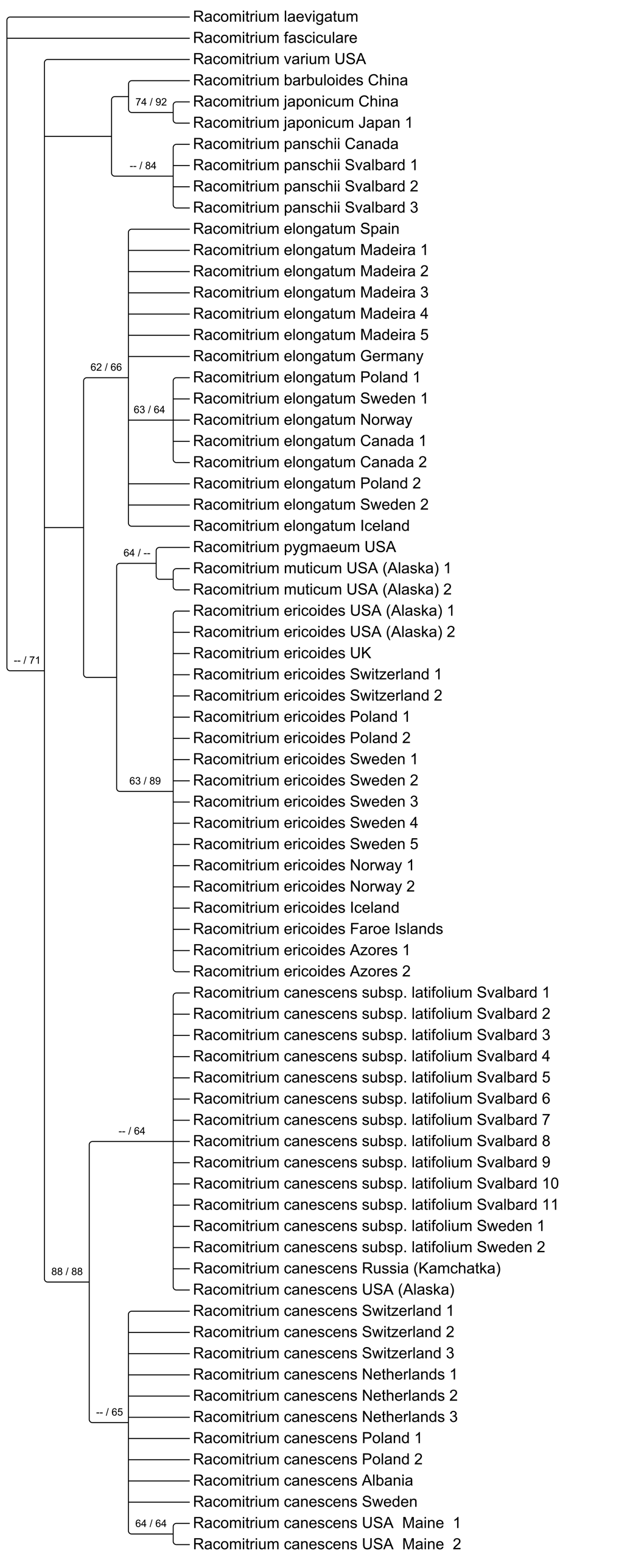

Supplement: Figure S3 — Maximum parsimony phylogenetic reconstruction of the Racomitrium canescens species complex based on plastid trnT-trnL sequences. Indels coded by simple indel coding were included. Bootstrap support values of the respective analyses without indels (before the slash) and with indels (after the slash) are indicated. (TIF) [file pone.0053134.s003.tif]

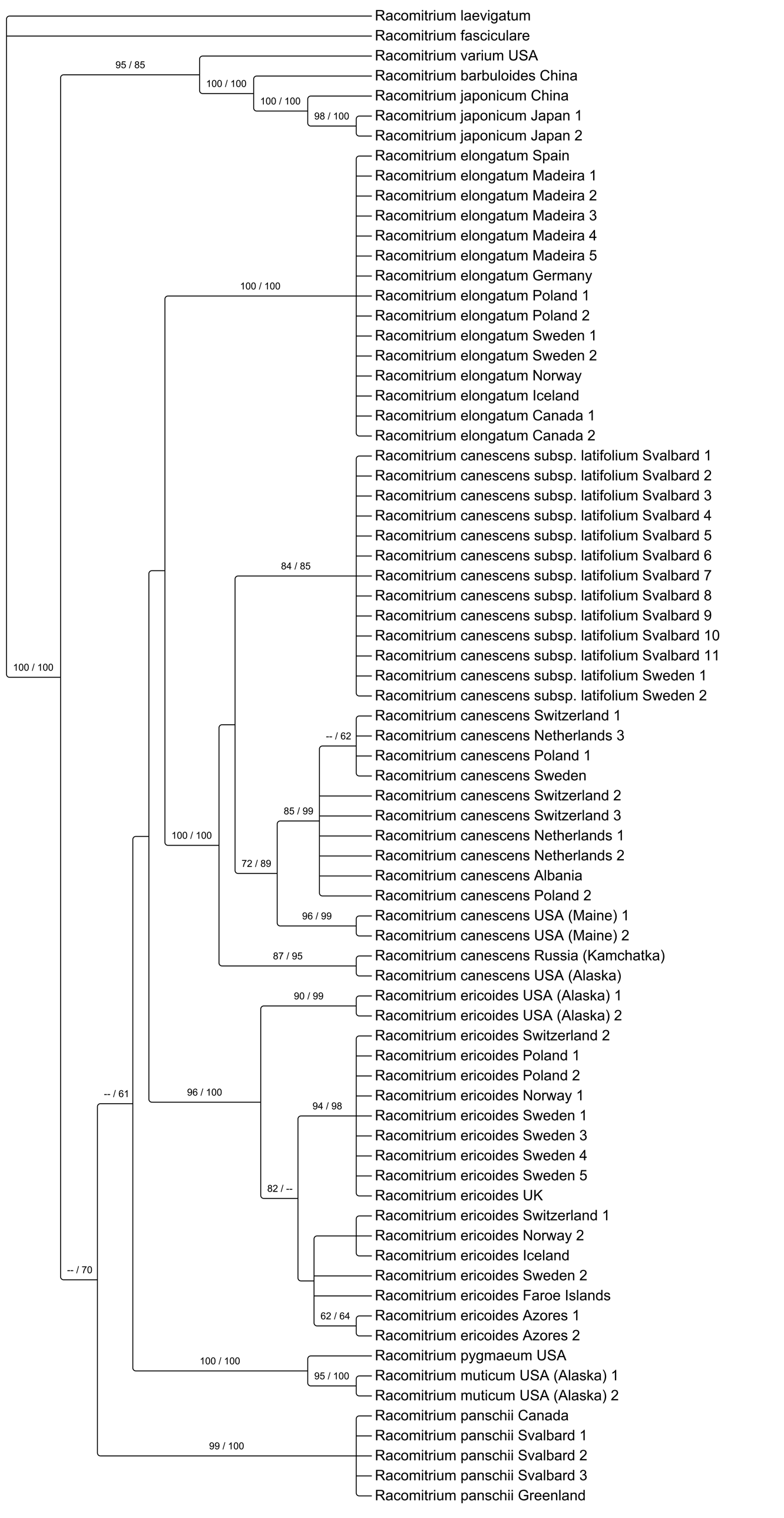

Supplement: Figure S4 — Maximum parsimony phylogenetic reconstruction of the Racomitrium canescens species complex based on nuclear ribosomal ITS sequences. Indels coded by simple indel coding were included. Bootstrap support values of the respective analyses without indels (before the slash) and with indels (after the slash) are indicated. (TIF) [file pone.0053134.s004.tif]

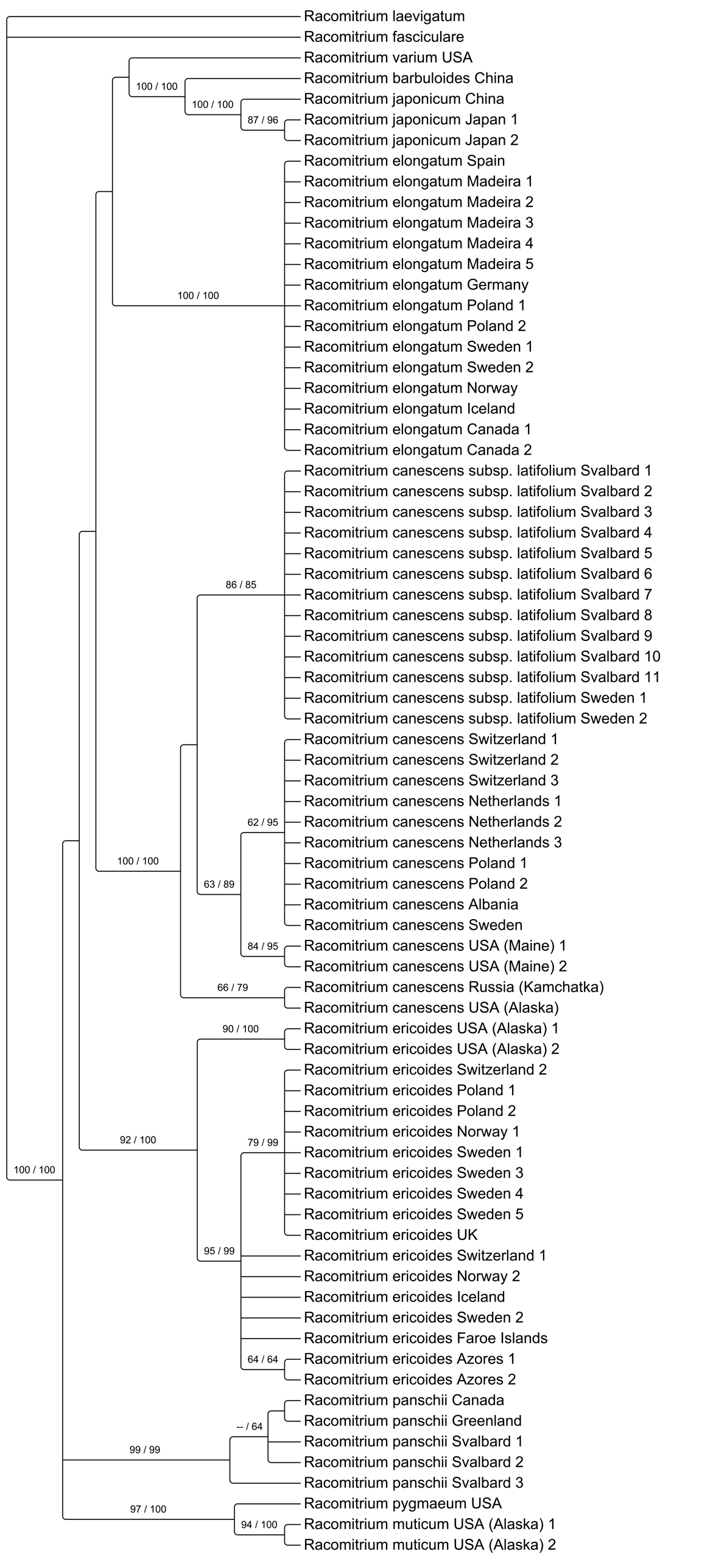

Supplement: Figure S5 — Maximum parsimony phylogenetic reconstruction of the Racomitrium canescens species complex based on nuclear ribosomal ITS1 sequences. Indels coded by simple indel coding were included. Bootstrap support values of the respective analyses without indels (before the slash) and with indels (after the slash) are indicated. (TIF) [file pone.0053134.s005.tif]

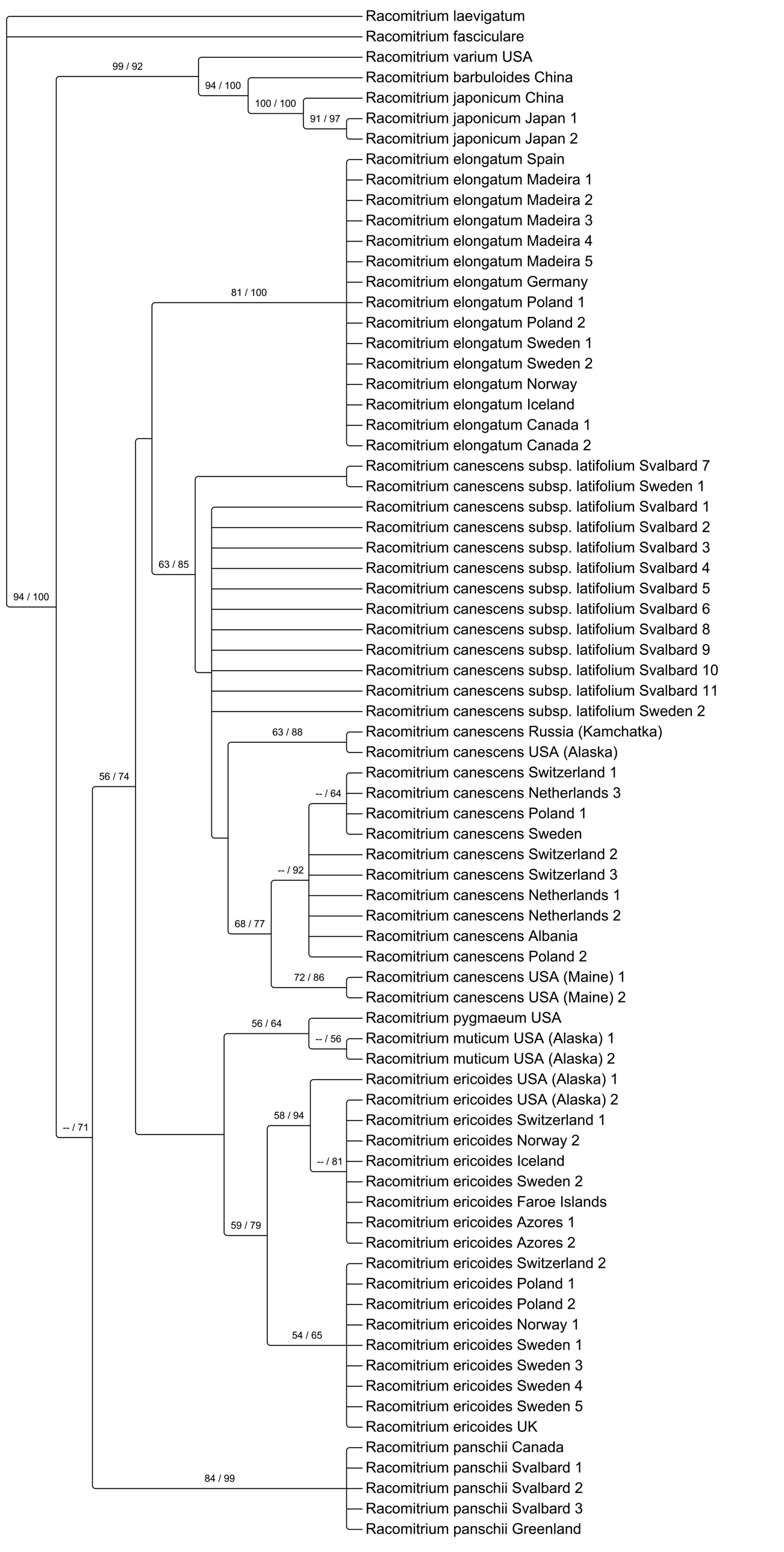

Supplement: Figure S6 — Maximum parsimony phylogenetic reconstruction of the Racomitrium canescens species complex based on nuclear ribosomal ITS2 sequences. Indels coded by simple indel coding were included. Bootstrap support values of the respective analyses without indels (before the slash) and with indels (after the slash) are indicated. (TIF) [file pone.0053134.s006.tif]
