# Supplementary material for: Molecular Species Delimitation in the Racomitrium canescens Complex (Grimmiaceae) and Implications for DNA Barcoding of Species Complexes in Mosses
Source: PLoS One. 2013 Jan 14;8(1):e53134. doi: 10.1371/journal.pone.0053134 (PMC3544804; doi:10.1371/journal.pone.0053134)
Supplement: Appendix S1 — Geographic origin (with numbering corresponding to Fig. 1 of the manuscript), voucher information and herbarium locations (in brackets), and GenBank accession numbers ( rps4-trnT-trnL , nrITS) of 70 Racomitrium specimens newly sequenced for the present study. (DOC) [file pone.0053134.s007.doc]

**Appendix S1.** Geographic origin (with numbering corresponding to Fig. 1 of the manuscript), voucher information and herbarium locations (in brackets), and GenBank accession numbers (*rps4-trnT-trnL*, nrITS) of 70 *Racomitrium* specimens newly sequenced for the present study.

*R. barbuloides* Cardot, China, Shevock 23044 (KRAM), JX069477, JX069483; *R. canescens* (Hedw.) Brid., Albania, van Zanten 04.06.203 (L), JX069612, JX069548; Netherlands 1, Kortselius 2008.11.0002 (L), JQ690732, JX069544; Netherlands 2, Kortselius 2008.11.0007 (L), JX069609, JX069545; Netherlands 3, L0857305 (L), JX069610, JX069546; Poland 1, B110440 (S), JX069611, JX069547; Poland 2, B110442 (S), JX069614, JX069550; Russia (Kamchatka), B119481 (S), JX069604, JX069539; Sweden, B122753 (S), JX069613, JX069549; Switzerland 1, G00128306 (G), JX069606, JX069541; Switzerland 2, G00110870 (G), JX069607, JX069542; Switzerland 3, G00110871 (G), JX069608, JX069543; USA (Alaska), B63648 (S), JX069605, JX069540; USA (Maine) 1, B142049 (S), JX069615, JX069551; USA (Maine) 2, Allen 24598A (L ex MO), JX069616, JX069552; *R. canescens* subsp. *latifolium* (Lange & C.E.O. Jensen) Frisvoll, Sweden 1, B122752 (S), JX069602, JX069537; Sweden 2, B143493 (S), JX069603, JX069538; Spitsbergen 1, Stech & Kruijer 08-284 (L), JX069591, JX069526; Spitsbergen 2, Stech & Kruijer 08-040 (L), JX069592, JX069527; Spitsbergen 3, Stech & Kruijer 08-296 (L), JX069593, JX069528; Spitsbergen 4, Stech & Kruijer 08-274 (L), JX069594, JX069529; Spitsbergen 5, Stech & Kruijer 08-281 (L), JX069595, JX069530; Spitsbergen 6, Stech & Kruijer 08-292 (L), JX069596, JX069531; Spitsbergen 7, Stech & Kruijer 08-280 (L), JX069597, JX069532; Spitsbergen 8, Stech & Kruijer 08-275 (L), JX069598, JX069533; Spitsbergen 9, Stech & Kruijer 08-291 (L), JX069599, JX069534; Spitsbergen 10, Stech & Kruijer 08-294 (L), JX069600, JX069535; Spitsbergen 11, Stech & Kruijer 09-061 (L), JX069601, JX069536; *R. elongatum* Ehrh. ex Frisvoll, Canada 1, B47385 (S), JX069568, JX069500; Canada 2, B47738 (S), JX069569, JX069501; Germany, L0857306 (L), JX069560, JX069492; Iceland, Schreuder-Sternermark 28 (L), JX069567, JX069499; Madeira 1, Stech 07-040 (L), JX069555, JX069487; Madeira 2, Stech 07-043 (L), JX069556, JX069488; Madeira 3, Stech 07-022 (L), JX06955, JX069489; Madeira 4, Stech 04-259 (L), JX069558, JX069490; Madeira 5, Pfeiffer 2000-50 (L), JX069559, JX069491; Norway, B63154 (S), JX069566, JX069498; Poland 1, B92411 (S), JX069562, JX069494; Poland 2, B95391 (S), JX069563, JX069495; Sweden 1, B11073 (S), JX069564, JX069496; Sweden 2, B31900 (S), JX069565, JX069497; *R. ericoides* (Brid.) Brid., Azores 1, Stech 08-460 (L), JX069589, JX069524; Azores 2, Stech 08-461 (L), JX069590, JX069525; Faroe Is., L0857304 (L), JX069588, JX069523; Iceland, Schreuder-Sternermark 17 (L), JX069586, JX069521; Norway 1, B63153 (S), JX069578, JX069513; Norway 2, B83904 (S), JX069585, JX069520; Sweden 1, B121453 (S), JX069579, JX069514; Sweden 2, B114338 (S), JX069587, JX069522; Sweden 3, B99982 (S), JX069580, JX069515; Sweden 4, B113755 (S), JX069581, JX069516; Sweden 5, B121453 (L ex S), JX069582, JX069517; Switzerland 1, G00128307 (G), JX069584, JX069519; Switzerland 2, Lang 20090804.3 (L), JX069577, JX069512; UK, Kruijer 2010.05.0004 (L), JX069583, JX069518; USA (Alaska) 1, B95132 (S), JX069575, JX069510; USA (Alaska) 2, B85164 (S), JX069576, JX069511; *R. japonicum* Dozy & Molk., China, Buck 23657 (KRAM), JX069478, JX069484; Japan 1, Mizutani 15454 (L), JX069553, JX069485; Japan 2, Mizutani 15628 (L), JX069554, JX069486; *R. muticum* (Kindb.) Frisvoll, USA (Alaska) 1, Schofield 113261 (KRAM), JX069481, JX069508; USA (Alaska) 2, B144567 (S), JX069574, JX069509; *R. panschii* (Müll. Hal.) Kindb., Canada, Allen 19833 (MA), JX069479, JX069502; Greenland, Hassel & Prestø 76 (L ex TRD), JX069573, JX069506; Spitsbergen 1, Stech & Kruijer 08-293 (L), JX069570, JX069503; Spitsbergen 2, Stech & Kruijer 09-001 (L), JX069571, JX069504; Spitsbergen 3, Stech & Kruijer 09-059 (L), JX069572, JX069505; *R. pygmaeum* Frisvoll, USA, Spence 1765 (KRAM), JX069480, JX069507; *R. varium* (Mitt.) A. Jaeger, USA, Shevock 21746 (KRAM), JX069476, JX069482.
